# DNA-based characterization of rays (Elasmobranchii: Batoidea) from Bangladesh using mitochondrial markers: Implications for conservation and management

**DOI:** 10.1371/journal.pone.0344352

**Published:** 2026-03-05

**Authors:** Mysha Mahjabin, Sujan Kumar Datta, Ahmed Farhan Labib, Sanjida Akhter, Durjoy Raha Antu, Md. Sagir Ahmed

**Affiliations:** 1 Advanced Fisheries and DNA Barcoding Laboratory, Department of Zoology, University of Dhaka, Dhaka, Bangladesh; 2 Florey Institute of Neuroscience and Mental Health, University of Melbourne, Victoria, Australia; 3 Department of Zoology, Jagannath University, Dhaka, Bangladesh; Universidade Federal do Rio de Janeiro, BRAZIL

## Abstract

Rays are an iconic group of chondrichthyan fishes, with many species currently threatened with extinction. Although conservation laws exist in Bangladesh to protect their population, lack of comprehensive law enforcement strategies together with commercial exploitation and habitat destruction resulted in population decline of many species nonetheless. One significant challenge to this conservation effort is a rapid and authentic species identification strategy, as traditional morphological diagnosis is hindered by frequent misidentification, especially when species are morphologically similar or when specimens are damaged or missing key features. The emergence of DNA barcoding technique can overcome this barrier, requiring only a small tissue sample for authentic identification. In the present study, this state-of-the-art technique has been employed for species identification of rays using three different mitochondrial marker gene, namely 16s rRNA, COI, and NADH2. A total of 94 new barcode sequences were generated, including 43 COI, 31 16S rRNA, and 20 NADH2 sequences, representing 23 ray species across 15 genera, 9 families, and 3 orders. Mean genetic distances varied across markers: for COI, 0.23 within species, 7.60 within genera, and 17.34 within families; for 16S rRNA, 0.04, 5.49, and 8.72, respectively; and for NADH2, 0.22, 13.52, and 20.72, respectively. Based on genetic divergence, barcode gap, and phylogenetic resolution, NADH2 proved to be a valuable alternative marker to COI for species-level identification. In contrast, 16s rRNA displayed the lowest divergence limiting its discriminatory power for species-level identification. Approximately 82.61% of our recorded species are categorized as different threatened categories (CR, EN, VU, NT) under the IUCN Global Red List. However, only 4 species are listed in CITES Appendix II for protection, leaving the majority of the ray species vulnerable for exploitation. Furthermore, several Schedule I and II species under Bangladesh Wildlife Act are openly traded in domestic market despite their supposed protection. This study highlights the urgent need to raise awareness among fishing communities and to strengthen measures against this illegal trade of ray species listed under national wildlife protection schedules.

## Introduction

Elasmobranchs (sharks, skates, and rays) are among the most imperiled large marine species, where 37% of them are at risk of extinction, with around 7.5% classified as Critically Endangered [1]. With slow reproductive cycle [2] and intense exploitation, such as by habitat destruction, and intentional and accidental bycatch, there has been a decline in the population across numerous elasmobranch species [3–7]. As such, there is an urgent need for effective conservation measures to prevent them from risk with extinction [4,8].

Rays, under the superorder Batoidea, are diverse group of cartilaginous fishes performing essential ecological functions including nutrient cycling and habitat modification [9,10]. Despite their wide distribution and commercial importance, little scientific attention has been given to these species over other vertebrates [5,11]. Currently, the total number of described ray species from Bangladesh is disputed varying from 25 to 58 due to lack of the intensive survey in the coastal region [12–14].

Under the Bangladesh Wildlife (Conservation and Security) Act, 2012 [15], a total of 14 threatened ray species were included in the Schedule I and 17 species in the Schedule II. However, increase in demand for ray meat and other derived products for both domestic consumption and international trade together with ineffective law enforcement strategy resulted in insufficient monitoring, control, and surveillance (MCS) mechanisms, leading to the population decline [13,16,17].

Identification of elasmobranchs in global trade through morphological diagnosis remains a challenge as harvested products are often processed, cut, or otherwise altered, making morphological traits cryptic or absent [18–22]. Moreover, traditional morphological keys are often limited by life stage, sex, or specimen preservation, making identification of rays especially challenging. Fortunately, emergence of DNA barcoding has overcome these limitations, allowing accurate species identification without requiring morphological traits [23–27]. This state-of-art technique has been particularly effective in trade monitoring, enabling the tracing of shark and ray products through unregulated market chains [19].

Few attempts were made on molecular characterization of rays from Bangladesh based on Cytochrome c oxidase subunit I (COI) marker [23,28]. The present study aims to evaluate the efficacy of three mitochondrial markers COI, 16S rRNA, and NADH dehydrogenase subunit 2 (NADH2); for ray identification, explore potential new species or cryptic records, and determine phylogenetic relationships among rays from northeastern Bay of Bengal.

## Materials and methods

### Sample collection and identification

Fish samples were collected from fish landing centers, local markets, and fishermen in the Cox’s Bazar, Chattogram, and Patuakhali regions between March 2021 and June 2025. A minimum of three specimens were obtained for each species; however, for rarely found species, only a single specimen was analyzed. The experimental design involved collecting dead specimens from fish markets and landing centers; therefore, the relevant institutional ethics committee deemed that ethical approval was not required for this study. Digital photographs were taken immediately after collection, and taxonomic identification was carried out based on established references [10,14,29]. Tissue samples were promptly excised from the specimens and preserved in 90% ethanol. These samples were then transported to the Advanced Fisheries and DNA Barcoding Laboratory, Department of Zoology, University of Dhaka, for further analysis. Voucher specimens were archived at the Dhaka University Zoology Museum (DUZM).

### DNA extraction, PCR amplification and sequencing

Approximately 20–40 mg of tissue was excised from the ventral region of each specimen using sterilized forceps and scissors after removing the ventral skin, and the fresh tissue was transferred to 1.5 mL microcentrifuge tubes, with a portion immediately processed for DNA extraction and the remainder preserved in 100% ethanol at −20 °C for future analyses. Genomic DNA was extracted using the Qiagen® DNeasy Blood and Tissue Kit (USA) following the manufacturer’s protocol, and DNA concentration and purity were measured with a NanoDrop™ spectrophotometer (Thermo Fisher Scientific). Three mitochondrial gene regions—cytochrome c oxidase subunit I (COI), 16S rRNA, and NADH dehydrogenase subunit 2 (NADH2) were amplified by polymerase chain reaction (PCR) using the primer pairs FishF2/FishR2 [30], 16Sar_F/16Sbr_R [31], and ILEM_F/ASNM_R [32], respectively. Each 25 μL PCR reaction contained 2 μL of DNA template, 12.5 μL of Taq polymerase master mix, 1 μL of each primer (10 μM), and 8.5 μL of nuclease-free water, and reactions were briefly centrifuged to mix. Amplification conditions consisted of an initial denaturation at 95 °C for 5 min; 41 cycles of 95 °C for 30 s, annealing at 48–54 °C (COI-52°C, 16S rRNA-48°C, and NADH2–54°C) for 30 s, and extension at 72 °C for 1 min; followed by a final extension at 72 °C for 5 min. PCR products were visualized on 1% agarose gels stained with Midori Green Advance dye, and only samples with clear bands were submitted for bidirectional Sanger sequencing.

### Bioinformatics analysis

Verified sequences were deposited in both the Barcode of Life Data System (BOLD Systems) [33] and NCBI GenBank (https://www.ncbi.nlm.nih.gov/). All sequences were aligned using MUSCLE [34], and genetic pairwise divergence was estimated with the Kimura 2-parameter (K2P) model [35] in BOLD. Intra- and interspecific genetic divergences were summarized as box-plot distributions in Microsoft Excel. Phylogenetic relationships for COI, 16S rRNA and NADH2 sequences were inferred using the Neighbor-Joining (NJ) method with gamma-distributed rates and bootstrap support based on 1000 replicates in MEGA 11 [36], and the resulting trees were visualized using iTOL v5 [37]. Operational Taxonomic Units (OTUs) were estimated using the REfined Single Linkage algorithm (RESL) [38] in BOLD to delineate closely related species based on COI sequences.

## Results

The study documented 23 species of rays belonging to 15 genera, 9 families, and 3 orders. Among them, 9 species (39.13%) fall under Endangered (EN), 3 species (13.04%) as Vulnerable (VU), 3 species (13.04%) as Near Threatened (NT), 4 species (17.39%) as Critically Endangered (CR), and 1 species (4.34%) as Data Deficient (DD), while 3 species (13.04%) remain Not Evaluated (NE) in the IUCN global Red List categories (Table 1). From these identified species, a total of 56 samples were taken for molecular study and generated 94 DNA barcode sequences, comprising of 43 COI, 31 16S rRNA, and 20 NADH2 sequences (Table 2). All sequences were deposited in GenBank (with accession numbers) and BOLD (with process IDs) (Table 2). The BOLD dataset was designated as “DS-RAYS” for subsequent analyses.

**Table 1:** Conservation Status of Ray Species in Bangladesh: IUCN Red List, CITES Listings, and Protection under the Wildlife (Conservation and Security) Act, 2012.

SL No	Order	Family	Species	Common Name	National protection*	CITES listing	IUCN status
1	Myliobatiformes	Aetobatidae	*Aetobatus ocellatus*	Ocellated Eagle Ray	Schedule II	–	EN (2023)
2		Dasyatidae	*Brevitrygon walga*	Bengal whipray	Not Protected	–	NT (2017)
3			*Hemitrygon bennettii*	Bennett’s Stingray	Not Protected	–	VU (2019)
4			*Himantura fava*	Honeycomb whipray	Not Protected	–	NE
5			*Himantura leoparda*	Leopard whipray	Schedule II	–	EN (2023)
6			*Himantura undulata*	Honeycomb whipray	Schedule II	–	EN (2020)
7			*Maculabatis gerrardi*	Sharp Nose Stingray	Schedule I	–	EN (2020)
8			*Maculabatis pastinacoides*	Round Whipray	Schedule II	–	EN (2020)
9			*Neotrygon indica*	Indian Ocean blue spotted Maskray	Not Protected	–	NE
10			*Neotrygon kuhlii*	Blue-spotted stingray	Not Protected	–	DD (2017)
11			*Pastinachus sephen*	Cowtail Stingray	Not Protected	–	NT (2017)
12			*Pateobatis bleekeri*	Bleeker’s Whipray	Schedule I	–	EN (2020)
13			*Pateobatis jenkinsii*	Jenkins’ Whipray	Schedule II	–	EN (2023)
14			*Pateobatis uarnacoides*	Whitenose Whipray	Schedule II	–	EN (2020)
15		Mobulidae	*Mobula mobular*	Spinetail Devil Ray	Schedule I	Appendix II	CR (2025)
16		Gymnuridae	*Gymnura poecilura*	Long-tailed butterfly ray	Schedule II	–	VU(2020)
17		Rhinopteridae	*Rhinoptera jayakari*	Oman Cownose Ray	Schedule I	–	EN (2020)
18			*Rhinoptera steindachneri*	Pacific Cownose Ray	Not Protected	Appendix II	NT (2019)
19	Torpediniformes	Narcinidae	*Narcine brunnea*	Brown numb ray	Not Protected	–	NE
20			*Narcine timlei*	Spotted Electric Ray	Schedule II	–	VU (2020)
21	Rhinopristiformes	Rhinobatidae	*Rhinobatos annandalei*	Annandale’s guitarfish	Schedule I	–	CR (2020)
22		Rhinidae	*Rhina ancylostomus*	Bowmouth guitarfish	Schedule I	Appendix II	CR (2018)
23		Glaucostegidae	*Glaucostegus granulatus*	Granulated guitarfish	Schedule I	Appendix II	CR (2022)

(EN Endangered; NT Near Threatened; VU Vulnerable; DD Data Deficient; LC Least Concern; NE-Not Evaluated).

* Wildlife (Conservation and Security) Act, 2012, Bangladesh; IUCN- International Union for Conservation of Nature; CITES- Convention on International Trade in Endangered Species of Wild Fauna and Flora.

**Table 2:** Sequences of Rays samples used in this study with their location and GPS coordinates.

Species Name	BOLD Process ID	GenBank Accession Number	BIN	Voucher ID	Place of Collection	GPS coordinates
**COI Sequences**						
*Neotrygon indica*	DUMBF087−23	OQ628387	BOLD:AAA5611	DUZM_MF_030B.4	Cox`s Bazar	21.24 N 91.76 E
*Neotrygon indica*	DUMBF095−23	OQ628395	BOLD:AAA5611	DUZM_MF_030B.5	Cox`s Bazar	21.24 N 91.76 E
*Neotrygon indica*	DUMBF079−23	OQ628379	BOLD:AAA5611	DUZM_MF_030B.3	Cox`s Bazar	21.24 N 91.76 E
*Neotrygon indica*	DUMBF083−23	OQ628383	BOLD:AAA5611	DUZM_MF_030B.5	Cox`s Bazar	21.24 N 91.76 E
*Neotrygon kuhlii*	GBGC15124−19	MN013424	BOLD:AAA5611	DUZM_MF_029	Cox`s Bazar	21.28 N 91.25 E
*Neotrygon kuhlii*	GBGC15125−19	MN013426	BOLD:AAA5611	DUZM_MF_029.2	Cox`s Bazar	21.28 N 91.25 E
*Hemitrygon bennettii*	DUMBF089−23	OQ628389	BOLD:AAC2114	DUZM_MF_028	Cox`s Bazar	21.24 N 91.76 E
*Gymnura poecilura*	DUMBF090−23	OQ628390	BOLD:ADP3982	DUZM_MF_027.6	Cox`s Bazar	21.24 N 91.76 E
*Gymnura poecilura*	DUMBF072−23	OQ628372	-	DUZM_MF_027.5	Cox`s Bazar	21.24 N 91.76 E
*Gymnura poecilura*	DUMBF076−23	OQ628376	BOLD:ADP3982	DUZM_MF_027.4	Cox`s Bazar	21.24 N 91.76 E
*Gymnura poecilura*	DUMBF080−23	OQ628380	BOLD:ADP3982	DUZM_MF_027.5	Cox`s Bazar	21.24 N 91.76 E
*Gymnura poecilura*	ANGBF47814−19	MH429307	BOLD:ADX7683	DUZM_MF_027.2	Cox’s Bazar	21.37 N 91.54 E
*Brevitrygon walga*	DUMBF091−23	OQ628391	BOLD:ADK1149	DUZM_MF_036.9	Cox`s Bazar	21.24 N 91.76 E
*Brevitrygon walga*	DUMBF092−23	OQ628392	BOLD:ADK1149	DUZM_MF_036.10	Cox`s Bazar	21.24 N 91.76 E
*Brevitrygon walga*	DUMBF093−23	OQ628393	BOLD:ADK1149	DUZM_MF_036.11	Cox`s Bazar	21.24 N 91.76 E
*Maculabatis gerrardi*	DUMBF070−23	OQ628370	BOLD:ADM5054	DUZM_MF_036B.14	Cox`s Bazar	21.24 N 91.76 E
*Maculabatis gerrardi*	DUMBF094−23	OQ628394	BOLD:ADM5054	DUZM_MF_036B.16	Cox`s Bazar	21.24 N 91.76 E
*Maculabatis gerrardi*	DUMBF096−23	OQ628396	BOLD:ADM5054	DUZM_MF_036B.17	Cox`s Bazar	21.24 N 91.76 E
*Maculabatis gerrardi*	DUMBF097−23	OQ628397	BOLD:ADM5054	DUZM_MF_036B.18	Cox`s Bazar	21.24 N 91.76 E
*Maculabatis gerrardi*	DUMBF085−23	OQ628385	BOLD:ADM5054	DUZM_MF_036B.15	Cox`s Bazar	21.24 N 91.76 E
*Maculabatis pastinacoides*	DUMBF077−23	OQ628377	BOLD:ACB2497	DUZM_MF_036B.2	Cox`s Bazar	21.24 N 91.76 E
*Maculabatis pastinacoides*	DUMBF081−23	OQ628381	BOLD:ACB2497	DUZM_MF_036B.3	Cox`s Bazar	21.24 N 91.76 E
*Maculabatis pastinacoides*	DUMBF084−23	OQ628384	BOLD:ACB2497	DUZM_MF_036B.4	Cox`s Bazar	21.24 N 91.76 E
*Maculabatis pastinacoides*	DUMBF100−23	OQ628400	BOLD:ACB2497	DUZM_MF_036B.5	Chattogram	22.33 N 91.85 E
*Himantura undulata*	DUMBF098−23	OQ628398	BOLD:AAF0692	DUZM_MF_036E.5	Chattogram	22.33 N 91.85 E
*Himantura leoparda*	DUMBF102−23	OQ628402	BOLD:AAB7831	DUZM_MF_036F	Cox`s Bazar	21.24 N 91.76 E
*Himantura fava*	DUMBF104−23	OQ628404	BOLD:AAF0692	DUZM_MF_036G	Cox`s Bazar	21.24 N 91.76 E
*Pateobatis uarnacoides*	DUMBF099−23	OQ628399	BOLD:AAC0562	DUZM_MF_036D.4	Cox`s Bazar	21.24 N 91.76 E
*Pateobatis uarnacoides*	ANGBF48218−19	MH230950	BOLD:AAC0562	DUZM_MF_036D	Cox`s Bazar	21.24 N 91.76 E
*Pateobatis uarnacoides*	ANGBF48219−19	MH230951	BOLD:AAC0562	DUZM_MF_036D.2	Cox`s Bazar	21.24 N 91.76 E
*Pateobatis uarnacoides*	ANGBF48220−19	MH230953	BOLD:AAC0562	DUZM_MF_036D.3	Cox`s Bazar	21.24 N 91.76 E
*Pateobatis jenkinsii*	ANGBF48204−19	MH230946	BOLD:ADX1467	DUZM_MF_036C	Cox`s Bazar	21.24 N 91.76 E
*Pateobatis bleekeri*	DUMBF101−23	OQ628401	BOLD:AFE0313	DUZM_MF_036D.5	Chattogram	22.33 N 91.85 E
*Aetobatus ocellatus*	DUMBF071−23	OQ628371	BOLD:ACE7605	DUZM_MF_040B.12	Cox`s Bazar	21.24 N 91.76 E
*Aetobatus ocellatus*	DUMBF073−23	OQ628373	BOLD:ACE7605	DUZM_MF_040B.14	Cox`s Bazar	21.24 N 91.76 E
*Pastinachus sephen*	DUMBF075−23	OQ628375	BOLD:ACB9560	DUZM_MF_037	Cox`s Bazar	21.24 N 91.76 E
*Rhinoptera jayakari*	DUMBF105−23	OP860900	BOLD:AAC7667	DUZM_MF_045B.2	Cox`s Bazar	21.24 N 91.76 E
*Narcine timlei*	GBMNB8625−20	MN083137	BOLD:ACS3597	DUZM_MF_050	Cox`s Bazar	21.29 N 91.78 E
*Narcine brunnea*	GBMNB8593−20	MN083105	BOLD:ADX2795	DUZM_MF_049.4	Cox`s Bazar	21.29 N 91.78 E
*Mobula mobular*	DUMBF103−23	OQ628403	BOLD:AAB8636	DUZM_MF_044	Cox`s Bazar	21.24 N 91.76 E
*Mobula mobular*	ANGBF47540−19	MH230952	BOLD:AAB8636	DUZM_MF_043	Cox’s Bazar	21.24 N 91.76 E
*Glaucostegus granulatus*	GBMNF42950−22	OM985901	BOLD:ADW5239	DUZM_MF_025.4	Cox’s Bazar	21.47 N 91.39 E
*Glaucostegus granulatus*	GBMNF42951−22	OM985902	BOLD:ADW5239	DUZM_MF_025.5	Cox’s Bazar	21.47 N 91.39 E
**16S rRNA Sequences**						
*Neotrygon indica*	DUMBF013–23	OQ619164		DUZM_MF_030B.5	Cox`s Bazar	21.24 N 91.76 E
*Brevitrygon walga*	DUMBF018–23	OQ619169		DUZM_MF_036.17	Cox`s Bazar	21.24 N 91.76 E
*Brevitrygon walga*	DUMBF007–23	OQ619158		DUZM_MF_036.12	Cox`s Bazar	21.24 N 91.76 E
*Brevitrygon walga*	DUMBF008–23	OQ619159		DUZM_MF_036.13	Cox`s Bazar	21.24 N 91.76 E
*Brevitrygon walga*	DUMBF009–23	OQ619160		DUZM_MF_036.14	Cox`s Bazar	21.24 N 91.76 E
*Brevitrygon walga*	DUMBF011–23	OQ619162		DUZM_MF_036.16	Cox`s Bazar	21.24 N 91.76 E
*Brevitrygon walga*	DUMBF010–23	OQ619161		DUZM_MF_036.15	Cox`s Bazar	21.24 N 91.76 E
*Maculabatis pastinacoides*	DUMBF005–23	OQ619156		DUZM_MF_036B.7	Cox`s Bazar	21.24 N 91.76 E
*Maculabatis pastinacoides*	DUMBF001–23	OQ619152		DUZM_MF_036B.6	Cox`s Bazar	21.24 N 91.76 E
*Maculabatis pastinacoides*	DUMBF019–23	OQ619170		DUZM_MF_036B.8	Chattogram	22.33 N 91.84 E
*Maculabatis pastinacoides*	DUMBF021–23	OQ619172		DUZM_MF_036B.9	Cox`s Bazar	21.24 N 91.76 E
*Maculabatis gerrardi*	DUMBF012–23	OQ619163		DUZM_MF_036B.19	Cox`s Bazar	21.24 N 91.76 E
*Maculabatis gerrardi*	DUMBF014–23	OQ619165		DUZM_MF_036B.20	Cox`s Bazar	21.24 N 91.76 E
*Maculabatis gerrardi*	DUMBF015–23	OQ619166		DUZM_MF_036B.21	Cox`s Bazar	21.24 N 91.76 E
*Himantura undulata*	DUMBF002–23	OQ619153		DUZM_MF_036E.6	Cox`s Bazar	21.24 N 91.76 E
*Himantura undulata*	DUMBF016–23	OQ619167		DUZM_MF_036E.7	Chattogram	21.24 N 91.76 E
*Himantura fava*	DUMBF026−23	OQ619177		DUZM_MF_036G.2	Cox`s Bazar	21.24 N 91.76 E
*Himantura leoparda*	DUMBF022–23	OQ619173		DUZM_MF_036F.2	Cox`s Bazar	21.24 N 91.76 E
*Pateobatis uarnacoides*	DUMBF003–23	OQ619154		DUZM_MF_036D.6	Cox`s Bazar	21.24 N 91.76 E
*Pateobatis uarnacoides*	DUMBF017–23	OQ619168		DUZM_MF_036D.7	Cox`s Bazar	21.24 N 91.76 E
*Pateobatis bleekeri*	DUMBF020–23	OQ619171		DUZM_MF_036D.8	Chattogram	22.33 N 91.84 E
*Pastinachus sephen*	DUMBF004–23	OQ619155		DUZM_MF_037.2	Cox`s Bazar	21.24 N 91.76 E
*Hemitrygon bennettii*	DUMBF006–23	OQ619157		DUZM_MF_028.2	Cox`s Bazar	21.24 N 91.76 E
*Mobula japanica*	DUMBF023−23	OQ619174		DUZM_MF_044.2	Cox`s Bazar	21.24 N 91.76 E
*Mobula japanica*	DUMBF024−23	OQ619175		DUZM_MF_044.3	Cox`s Bazar	21.24 N 91.76 E
*Mobula mobular*	DUMBF025−23	OQ619176		DUZM_MF_044B.3	Cox`s Bazar	21.24 N 91.76 E
*Glaucostegus granulatus*	DUMBF106−25	OM760505		DUZM_MF_025.4	Cox`s Bazar	21.47 N 91.39 E
*Glaucostegus granulatus*	DUMBF107−25	OM760506		DUZM_MF_025.5	Cox`s Bazar	21.47 N 91.39 E
*Rhinobatos annandalei*	DUMBF108−25	PZ059839		DUZM_MF_024	Cox`s Bazar	21.47 N 91.39 E
*Rhina ancylostomus*	DUMBF109−25	MW514053		DUZM_MF_23S	St. Martin’s Island	20.60 E 92.29 E
*Rhinoptera steindachneri*	DUMBF110−25	OR818427		DUZM_MF_045B	Cox`s Bazar	21.23 N 91.75 E
**NADH2**						
*Maculabatis pastinacoides*	DUMBF001–23			DUZM_MF_036B.6	Cox`s Bazar	21.24 N 91.76 E
*Maculabatis pastinacoides*	DUMBF019–23			DUZM_MF_036B.8	Chattogram	22.33 N 91.84 E
*Maculabatis pastinacoides*	DUMBF021–23			DUZM_MF_036B.9	Cox`s Bazar	21.24 N 91.76 E
*Maculabatis pastinacoides*	DUMBF077−23			DUZM_MF_036B.2	Cox`s Bazar	21.24 N 91.76 E
*Maculabatis gerrardi*	DUMBF012–23			DUZM_MF_036B.19	Cox`s Bazar	21.24 N 91.76 E
*Maculabatis gerrardi*	DUMBF014–23			DUZM_MF_036B.20	Cox`s Bazar	21.24 N 91.76 E
*Maculabatis gerrardi*	DUMBF015–23			DUZM_MF_036B.21	Cox`s Bazar	21.24 N 91.76 E
*Himantura leoparda*	DUMBF022–23			DUZM_MF_036F.2	Cox`s Bazar	21.24 N 91.76 E
*Himantura undulata*	DUMBF098−23			DUZM_MF_036E.5	Chattogram	22.33 N 91.85 E
*Himantura undulata*	DUMBF002–23			DUZM_MF_036E.6	Cox`s Bazar	21.24 N 91.76 E
*Pateobatis bleekeri*	DUMBF020–23			DUZM_MF_036D.8	Chattogram	22.33 N 91.84 E
*Pateobatis uarnacoides*	DUMBF003–23			DUZM_MF_036D.6	Cox`s Bazar	21.24 N 91.76 E
*Pateobatis uarnacoides*	DUMBF099−23			DUZM_MF_036D.4	Cox`s Bazar	21.24 N 91.76 E
*Brevitrygon walga*	DUMBF018–23			DUZM_MF_036.17	Cox`s Bazar	21.24 N 91.76 E
*Brevitrygon walga*	DUMBF007–23			DUZM_MF_036.12	Cox`s Bazar	21.24 N 91.76 E
*Brevitrygon walga*	DUMBF008–23			DUZM_MF_036.13	Cox`s Bazar	21.24 N 91.76 E
*Brevitrygon walga*	DUMBF010–23			DUZM_MF_036.15	Cox`s Bazar	21.24 N 91.76 E
*Hemitrygon bennettii*	DUMBF089−23			DUZM_MF_028	Cox`s Bazar	21.24 N 91.76 E
*Pastinachus sephen*	DUMBF004–23			DUZM_MF_037.2	Cox`s Bazar	21.24 N 91.76 E
*Neotrygon indica*	DUMBF013–23			DUZM_MF_030B.5	Cox`s Bazar	21.24 N 91.76 E

BIN-Barcode Index Number; BINs are COI-specific, so other markers are not included in BIN-based analyses.

### Cytochrome c oxidase subunit I (COI) gene

**Fig 1:**
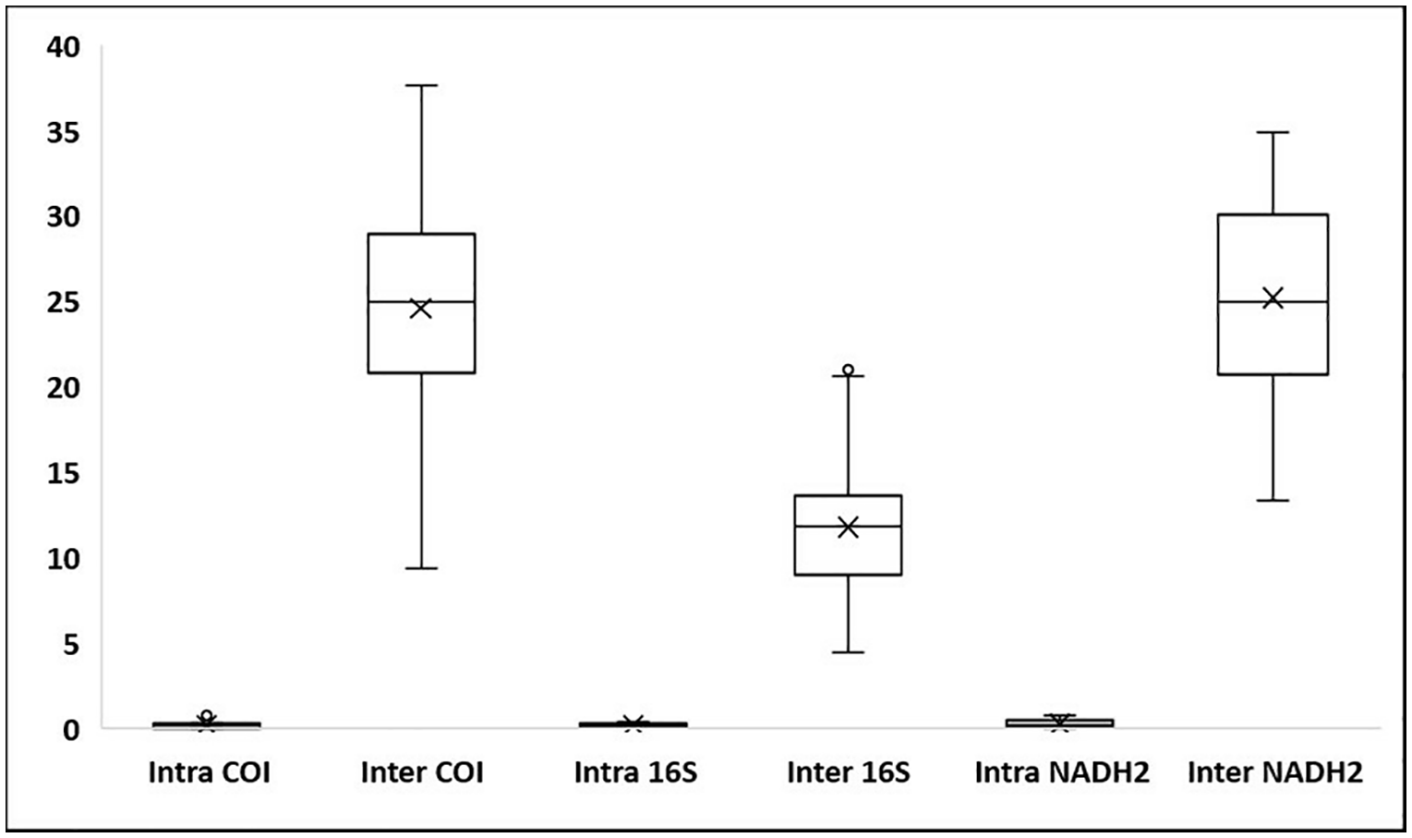
The box plot distribution of genetic divergence (K2P %) within and between species of rays based on COI, 16S rRNA and NADH2 genes.

**Fig 2:**
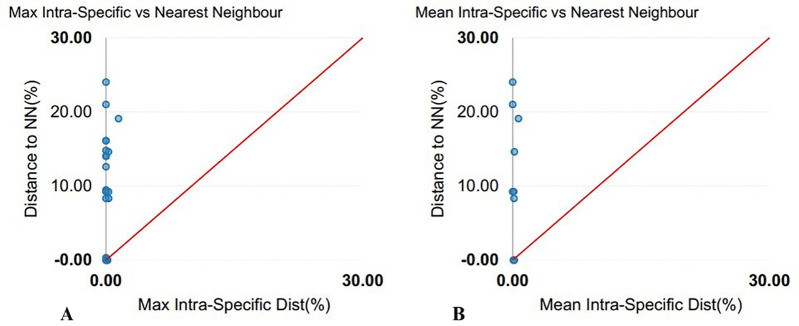
Genetic distance analysis of COI sequences obtained from BOLD using the Kimura 2-parameter (K2P) model. (A) Maximum intra-specific distance vs. nearest-neighbor distance, and (B) Mean intra-specific distance vs. nearest-neighbor species based on sequence divergence.

**Fig 3:**
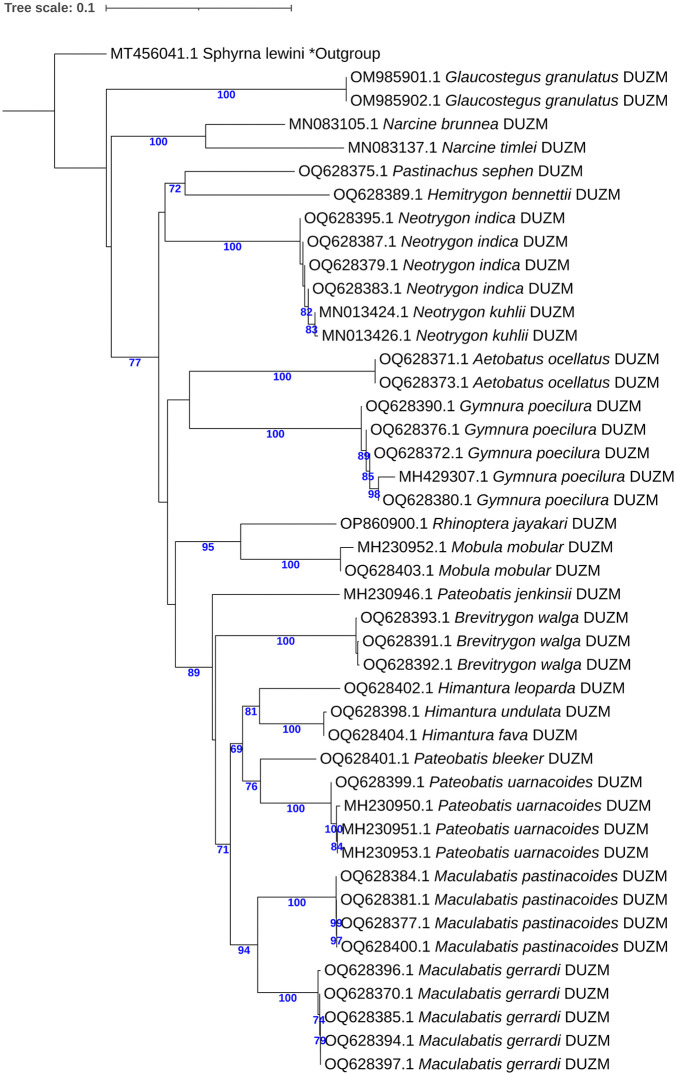
Neighbor-Joining (NJ) tree of ray species based on COI sequences using Kimura 2-parameter (K2P) distances. DUZM indicates species included in the present study, and * denotes the outgroup species.

The COI sequences ranged from 522 to 694 bp in length (mean = 666 bp), with 93% exceeding 600 bp. No stop codons, insertions, or deletions were detected. Average nucleotide frequencies were A: 24.89 ± 0.20%, T: 30.40 ± 0.25%, G: 17.36 ± 0.14%, and C: 27.35 ± 0.30%, resulting in an overall AT content of 55.29% and GC content of 44.71%. GC content across codon positions averaged 55.04 ± 0.26% (first), 42.61 ± 0.06% (second), and 36.48 ± 0.98% (third). Mean genetic distances were 0.23 ± 0.01 within species, 7.60 ± 0.13 within genera, and 17.34 ± 0.01 within families (Table 3), visualized by box plots (Fig 1). Sequence divergence across taxonomic levels, based on 43 COI sequences, revealed 20 operational taxonomic units (OTUs) (Table 4). The mean congeneric species distance was 33-fold higher than the mean conspecific individual distance, yielding a barcode gap of 8.62. Scatterplots illustrated the overlap of maximum and mean intraspecific distances with nearest-neighbor interspecific distances (Fig 2). The mean nearest-neighbor distance, estimated under the K2P model, was 10.08 ± 0.35 (Table 3). A Neighbor-Joining (NJ) phylogeny of 44 COI sequences (43 from this study and 1 from GenBank) showed conspecific individuals clustering together into well-supported clades (Fig 3).

**Table 3:** Genetic divergence (K2P Distance %) within species, genera and family) for COI, 16S rRNA and NADH2 gene.

Label	16S rRNA						COI						NADH2					
	n	Comparisons	Min Dist	Mean Dist	Max Dist	SE Dist	n	Comparisons	Min Dist	Mean Dist	Max Dist	SE Dist	n	Comparisons	Min Dist	Mean Dist	Max Dist	SE Dist
Within Species	21	28	0.00	0.04	0.34	0.00	31	44	0.00	0.23	1.46	0.01	15	17	0.00	0.22	0.82	0.01
Within Genus	17	21	0.00	5.49	12.24	0.14	27	37	0.00	7.60	15.17	0.13	13	16	11.77	13.52	14.83	0.06
Within Family	23	208	5.56	8.72	14.54	0.01	31	313	11.25	17.34	24.38	0.01	20	157	14.76	20.72	27.97	0.02

n-Number of sequences; Min-Minimum; Max-Maximum; Dist-Distance; SE- Standard Error.

**Table 4:** Distribution of sequence divergence at each taxonomic level (Cluster Sequence Result, Sequences: 43, OTUs: 20).

OTU	Average Distance	Max Distance	Taxon	Count	Distance to NN
OTU-1	0.15	0.15	*Mobula mobular*	2	11.378204
OTU-2	0.67	1.46	*Gymnura poecilura*	5	16.666668
OTU-3	0.0	0.0	*Pateobatis jenkinsii*	1	12.908498
OTU-4	0.15	0.31	*Pateobatis uarnacoides*	4	7.7826724
OTU-5	0.12	0.19	*Neotrygon kuhlii*	2	14.992722
OTU-6	0.12	0.19	*Neotrygon indica*	4	14.992722
OTU-7	0.0	0.0	*Narcine brunnea*	1	12.721416
OTU-8	0.0	0.0	*Narcine maculata*	1	12.721416
OTU-9	0.0	0.0	*Glaucostegus granulatus*	2	20.20202
OTU-10	0.12	0.29	*Maculabatis gerrardi*	5	8.550724
OTU-11	0.0	0.0	*Aetobatus ocellatus*	2	18.061674
OTU-12	0.0	0.0	*Pastinachus sephen*	1	14.347826
OTU-13	0.0	0.0	*Maculabatis pastinacoides*	4	8.550724
OTU-14	0.0	0.0	*Hemitrygon bennettii*	1	14.347826
OTU-15	0.19	0.29	*Brevitrygon walga*	3	13.011696
OTU-16	0.0	0.0	*Himantura undulata*	1	8.479532
OTU-17	0.0	0.0	*Himantura fava*	1	8.479532
OTU-18	0.0	0.0	*Himantura leoparda*	1	8.702065
OTU-19	0.0	0.0	*Pateobatis bleekeri*	1	7.7826724
OTU-20	0.0	0.0	*Rhinoptera jayakari*	1	11.378204

NN- Nearest-Neighbor; Count-Number of sequences.

### 16S rRNA gene

**Fig 4:**
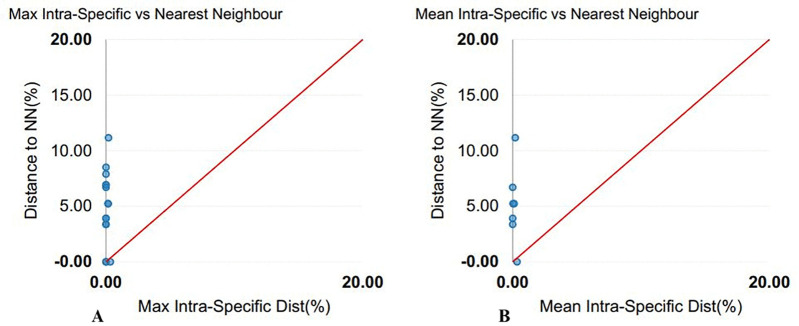
Genetic distance analysis of 16S rRNA sequences obtained from BOLD using the Kimura 2-parameter (K2P) model. (A) Maximum intra-specific distance vs. nearest-neighbor distance, and (B) Mean intra-specific distance vs. nearest-neighbor species based on sequence divergence.

**Fig 5:**
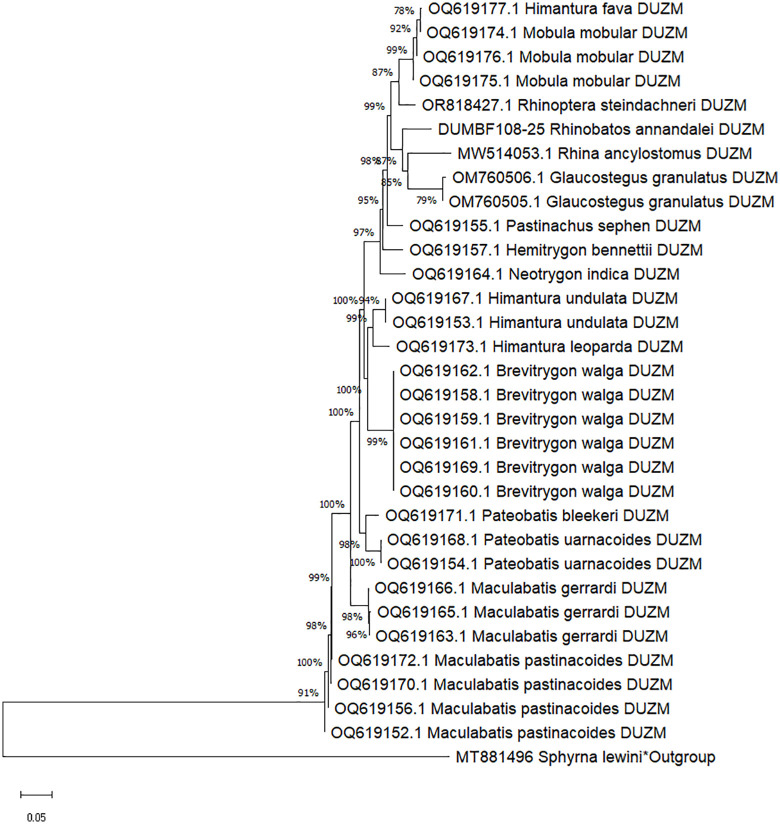
Neighbor-Joining (NJ) tree of ray species based on 16SrRNA sequences using Kimura 2-parameter (K2P) distances. DUZM indicates species included in the present study, and * denotes the outgroup species.

The 16S rRNA sequences ranged from 514 to 691 bp (mean = 622 bp), with 78% exceeding 600 bp. Average nucleotide composition was A: 31.37 ± 0.16%, T: 27.38 ± 0.15%, C: 21.97 ± 0.21%, and G: 19.28 ± 0.12%. The AT content (58.75%) was higher than the GC content (41.25 ± 0.27%). Genetic distances averaged 0.04 ± 0.00 within species, 5.49 ± 0.14 within genera, and 8.72 ± 0.01 within families (Table 3; Fig 1). The barcode gap was pronounced, with congeneric species distances 137-fold higher than conspecific individual distances (gap = 4.05). The mean nearest-neighbor distance (K2P model) was 4.88. Scatterplots of intra- versus interspecific distances are shown in Fig 4. The NJ tree generally grouped conspecific individuals together, however, several deeper nodes showed weak bootstrap support and some higher-level relationships were unresolved or incongruent with established phylogenies (Fig 5).

### NADH dehydrogenase subunit 2 (NADH2)

**Fig 6:**
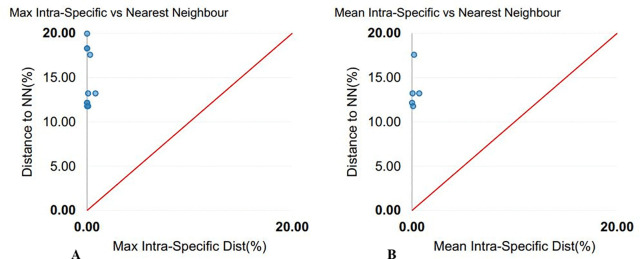
Genetic distance analysis of NADH2 sequences obtained from BOLD using the Kimura 2-parameter (K2P) model. (A) Maximum intra-specific distance vs. nearest-neighbor distance, and (B) Mean intra-specific distance vs. nearest-neighbor species based on sequence divergence.

**Fig 7:**
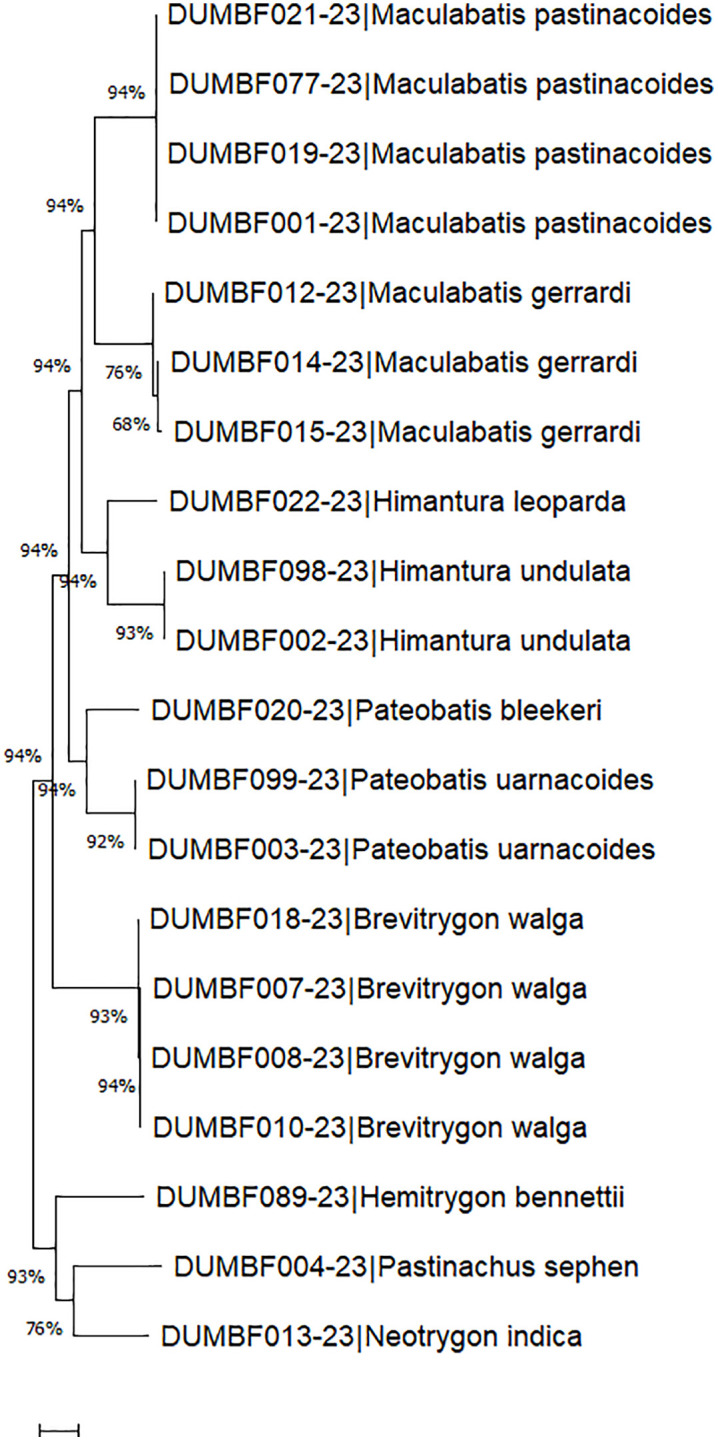
Neighbor-Joining (NJ) tree of ray species based on NADH2 sequences using Kimura 2-parameter (K2P) distances. DUZM indicates species included in the present study, and * denotes the outgroup species.

NADH2 sequences ranged from 789 to 1020 bp (mean = 989 bp), with 85% exceeding 1000 bp. No stop codons, insertions, or deletions were detected. Average nucleotide composition was A: 31.00 ± 0.12%, T: 27.51 ± 0.18%, G: 9.72 ± 0.12%, and C: 31.77 ± 0.27%, corresponding to an AT content of 58.51% and GC content of 41.49%. GC content by codon position averaged 45.49 ± 0.26% (first), 41.86 ± 0.21% (second), and 37.11 ± 0.64% (third). Mean genetic distances were 0.22 ± 0.01 within species, 13.52 ± 0.06 within genera, and 20.72 ± 0.02 within families (Table 3; Fig 1). The mean congeneric species distance was 61-fold higher than the mean conspecific individual distance, with a barcode gap of 12.58. Scatterplots comparing intra- and interspecific distances are shown in Fig 6. The mean nearest-neighbor distance, estimated with the K2P model, was 14.84 ± 0.31. An NJ tree of 21 NADH2 sequences (20 from this study and 1 from GenBank) grouped individuals by species into distinct clades (Fig 7).

## Discussion

### Species diversity and status

The study represents the first comprehensive molecular characterization of ray species from Bangladesh, documenting 23 species across 15 genera, 9 families, and 3 orders highlighting the ecological significance of Bangladeshi waters for the habitat of rays. According to the IUCN Global Red List [1], 82.61% of our recorded species are classified under different threatened categories (CR, EN, VU, NT), with *Mobula mobular, Rhinobatos annandalei, Rhina ancylostomus* and *Glaucostegus granulatus* listed as Critically Endangered (Table 1). At the national level under the Wildlife (Conservation and Security) Act, 2012 [15], 7 species (30.4%) are recognized as Schedule I (highest protection), 8 species (34.8%) as Schedule II, leaving 8 species (34.8%) without any protection level, well corresponding to the classification under the IUCN Global Red List. Consequently, more than two-thirds of ray species either receive lower protection or no protection at all, further amplifying conservation risks. Moreover, despite their legal protection status, many Schedule I and II species continue to be regularly landed and openly traded in domestic markets. This enforcement gap indicates that, in practice, the national wildlife act is insufficient to prevent exploitation. On top of this, CITES Appendix II includes only 4 species (17.4%) for protection, further leaving the majority of species vulnerable to exploitation and trade [39].

This mismatch among IUCN threat categories, national legislation, and CITES trade regulations highlights a critical conservation gap. Many species assessed as globally threatened remain unprotected in Bangladesh, underscoring the urgent need for stronger legal safeguards, stricter enforcement, and integration of molecular tools for monitoring and regulation.

### Genetic divergence patterns

A total of 94 new mitochondrial sequences of which 43 COI, 31 16S rRNA, and 20 NADH2 were generated and deposited in GenBank and BOLD (Table 2), significantly expanding reference libraries for elasmobranchs. Sequence analysis of these markers demonstrated high species-level resolution for rays. Under the K2P model, mean genetic distances showed clear taxonomic stratification, with COI displaying 0.23% divergence within species, 7.60% within genera, and 17.34% within families; 16S rRNA showing 0.04%, 5.49%, and 8.72% at the respective levels; and NADH2 showing 0.22%, 13.52%, and 20.72% (Fig 1 and Table 3). These genetic divergence values for COI and NADH2 align with previous elasmobranch studies [23,26,40]. There were 33-fold, 137-fold, and 61-fold more difference among congeneric species than conspecific individuals for COI, 16S rRNA and NADH2, respectively, indicating their reliable discriminating capability for species identification. Both COI and NADH2 displayed appreciable barcode gaps of 8.62 and 12.58 respectively (Fig 1), further demonstrating their strength for clear separation between intra- and interspecific genetic differences – a finding in line with previous elasmobranch studies from Australia, Indonesia, and India [41–43]. Indeed, Neighbor-Joining trees reinforced these findings, clustering conspecific individuals into well-supported clades and confirming morphological identifications (Figs 3, 5 and 7). By contrast, the 16S rRNA marker, on the other hand, showed relatively lower barcode gap value of 4.05, indicating a comparatively limited capacity for species-level discrimination.

### Efficacy of COI, 16S rRNA, and NADH2 markers

The comparative performance of the three mitochondrial markers revealed important differences in their phylogenetic resolution (Figs 3, 5 and 7). The COI gene demonstrated to be the most informative marker, consistently yielding high bootstrap support and forming monophyletic clades with clear species boundaries (Fig 3). Its discriminatory performance was particularly effective within genera such as *Maculabatis, Pateobatis,* and *Neotrygon*. These findings were consistent with previous studies that had established COI as a reliable DNA barcode marker for elasmobranchs [23,25,40,43].

In contrast, the 16S rRNA gene showed the weakest phylogenetic performance, with low bootstrap support and several unresolved or paraphyletic taxa (Fig 5). Importantly, the recovered topology failed to reflect accepted higher-level batoid relationships, as Rhinopristiformes was placed as a derived clade within Myliobatiformes, contradicting well-supported phylogenetic frameworks [29]. This indicates that, in the present dataset, the conserved nature of the 16S rRNA gene did not provide sufficient phylogenetic signal even for reliable higher-level placement. Consequently, 16S rRNA lacked the resolution necessary for both deep and shallow phylogenetic inference, limiting its utility for robust taxonomic and evolutionary interpretation in this study.

NADH2 provided an intermediate level of resolution compared to COI and 16S rRNA. The amplification rate of this marker was found to be relatively higher than the other two markers. It successfully resolved multiple species with strong bootstrap support, but taxa such as *Neotrygon indica* and *Himantura* species showed weaker resolution perhaps due to limited sequences in our studies (Fig 7). As such, we can reasonably expect NADH2 to be a valuable alternative marker to COI for species-level identification, which is consistent with previous findings [26].

The K2P distance patterns further clarify these differences. Intraspecific divergence was lowest for 16S rRNA (0.04%) and moderate for COI (0.23%), and NADH2 (0.22%), indicating that COI and NADH2 maintain species stability while enabling discrimination. At higher taxonomic levels, NADH2 exhibited the greatest divergence (13.52% within genera, 20.72% within families) followed by COI (7.60%, 17.34%). This demonstrated NADH2 to possess sufficient discriminatory power for species identification, with the added advantage of a relatively high amplification success rate, whereas COI provides the best balance across taxonomic levels- being sufficiently variable for species identification yet stable enough for broader resolution. In contrast, 16S rRNA displayed lower divergences (5.49%, 8.72%), reflecting its conserved nature, limiting its species level identification performance.

### Conservation and policy implications

Species identification in processed body parts is often difficult or impossible due to the absence of diagnostic features, further complicated by the use of non-taxonomic trade names that may apply to multiple morphologically similar species. The integration of molecular evidence with conservation assessments highlights the urgent need to strengthen protective measures for rays in Bangladesh. National legislation should be revised to include a greater proportion of threatened species, particularly those recognized by the IUCN as endangered or critically endangered. Expansion and/or improvement of CITES listings would further regulate international trade and help in reducing exploitation pressures. Beyond legislation, molecular tools should be incorporated into fisheries monitoring and enforcement, enabling rapid and accurate species identification at landing sites and in trade networks. Such integration would enhance conservation outcomes while supporting sustainable fisheries management. Finally, lack of public awareness and lenient enforcement of law on illegal, Unreported and Unregulated (IUU) fishing practices by the artisanal fishers leads to indiscriminate exploitation of the juveniles and adult rays in the coastal waters of Bangladesh. This factors also facilitate the illegal trade of endangered ray species. Hence, this study strongly recommends raising awareness among the fishers’ community and preventing illegal trade of ray species listed in the National Schedules and Appendices I & II of CITES.

## Conclusions

While this study establishes a solid molecular baseline for the diversity of ray species in Bangladesh, further research is needed to monitor population structure, connectivity, and temporal trends using genomic approaches. Coupling barcoding with ecological surveys, fisheries data, and socio-economic assessments will provide a more holistic framework for conservation planning. Focused research on domestic and international ray trade (Myliobatidae, Dasyatidae, and Glaucostegidae) in collaboration with local fishermen is urgently needed for elasmobranch conservation, as it remains overshadowed by the global emphasis on shark fin trade.
